# A benchmark of computational CRISPR-Cas9 guide design methods

**DOI:** 10.1371/journal.pcbi.1007274

**Published:** 2019-08-29

**Authors:** Jacob Bradford, Dimitri Perrin

**Affiliations:** School of Electrical Engineering and Computer Science, Queensland University of Technology, Brisbane, Queensland, Australia; University of Calgary, CANADA

## Abstract

The popularity of CRISPR-based gene editing has resulted in an abundance of tools to design CRISPR-Cas9 guides. This is also driven by the fact that designing highly specific and efficient guides is a crucial, but not trivial, task in using CRISPR for gene editing. Here, we thoroughly analyse the performance of 18 design tools. They are evaluated based on runtime performance, compute requirements, and guides generated. To achieve this, we implemented a method for auditing system resources while a given tool executes, and tested each tool on datasets of increasing size, derived from the mouse genome. We found that only five tools had a computational performance that would allow them to analyse an entire genome in a reasonable time, and without exhausting computing resources. There was wide variation in the guides identified, with some tools reporting every possible guide while others filtered for predicted efficiency. Some tools also failed to exclude guides that would target multiple positions in the genome. We also considered two collections with over a thousand guides each, for which experimental data is available. There is a lot of variation in performance between the datasets, but the relative order of the tools is partially conserved. Importantly, the most striking result is a lack of consensus between the tools. Our results show that CRISPR-Cas9 guide design tools need further work in order to achieve rapid whole-genome analysis and that improvements in guide design will likely require combining multiple approaches.

## Author summary

Modern genome engineering technologies provide unprecedented methods for DNA modifications. CRISPR-based systems generate a high level of interest in the community due to their effectiveness and relative simplicity. However, the design of the guides they use to target specific regions is not trivial. Researchers need to both maximise the likelihood of making the desired modification and minimise the risk of undesired, off-target changes. To assist researchers in making better informed decisions, a number of software tools have been developed to assist with guide design. Here, we analyse the performance of 18 design tools for CRISPR-Cas9 guides. We evaluate each tool based on their runtime, their computational requirements, and the output they generate. We benchmarked tools on datasets of increasing size (to evaluate their scalability) as well as on guides for which experimental data is available. Our results show that there is little consensus between the tools and that improvements in guide design will likely require combining multiple approaches.

This is a *PLOS Computational Biology* Benchmarking paper.

## Introduction

Wild-type CRISPR (Clustered Regularly Interspaced Short Palindromic Repeats) is found in archaea and bacteria, and acts as an adaptable immune system [1]. CRISPR is able to provide a method of immunity via three steps [2]: (i) a DNA snippet from an invading phage is obtained and stored within the CRISPR array, making a memory of past viral infection; (ii) the CRISPR region is expressed and matured to produce duplicates of previously obtained DNA snippets (or *guides*); (iii) in the case of *S. pyogenes* Cas9 (SpCas9), a guide binds with a SpCas9 nuclease to enable site-specific cleavage due to guide-DNA homology. This last step provides immunity to the host cell and is also the mechanism for CRISPR to be used in a genome engineering context, where a synthetic guide is supplied. CRISPR-based systems have been used for a number of such applications [3–5]. However, guide design is not trivial, as the efficiency and specificity of guides are crucial factors. For this reason, computational techniques are employed to identify and evaluate candidate CRISPR-Cas9 guides.

Here, we analyse 18 CRISPR-Cas9 guide design methods to evaluate whether they are adequate for rapid whole-genome analysis, and potentially whether combining approaches would achieve a solution of better quality. The available tools can be categorised based on algorithmic approach (i.e. procedural or via models trained using experimental data); however, when constructing the tool-set, we considered various factors: (i) whether the source code was easily obtained, (ii) is the installation process straight-forward, (iii) the tool simply does not provide a wrapper for performing a regular expression and (iv) the guide length and PAM sequence can be customised. In our analysis, we consider not only their output (i.e. which targets they identified), but also their ability to process whole genomes in a reasonable time. This is especially important for large genomes, such as some flowering plants. It is also a crucial feature for some applications such as studies of complex pathways or functions that require targeting multiple genes (e.g. sleep [6–8]) or producing whole-genome maps [9].

### Tool review

We selected 18 guide design tools that are released under an open-source license and report candidate guides for the *Streptococcus pyogenes*-Cas9 (SpCas9) nuclease; these tools are listed in Table 1. The most recent version was used for each tool, see Supplementary data for details.

**Table 1:** Tools selected in this study.

Tool name	Source	Rules	Specificity	Efficiency	Approach	Language	Bulge Support
CasFinder [32]	Tool website		List		Procedural	Perl	
CHOPCHOP [33]	BitBucket	GC%, FA	List	Filter, score	ML	Python	
sgRNACas9 [34]	SourceForge	GC%	Filter		Procedural	Perl	
GT-Scan [35]	Tool website	GC%	List		Procedural	Python	
CCTop [36]	BitBucket	GC%, FA	Score, list	Score	Procedural	Python	
SSC [31]	SourceForge			Score	ML	C	
CRISPR-ERA [37]	By request	PolyT, GC%	Score, list	Score	Procedural	Perl	
WU-CRISPR [38]	GitHub	PolyU, GC%, SSE	Filter	Score, filter	ML	Perl	
Cas-Designer [39]	Tool website	PolyT, GC%, FA	List	Score	Procedural	Python	✓
mm10db [6]	GitHub	PolyT, GC%, SSE, FA	Score, filter	Filter	Procedural	Python/C	
CT-Finder [40]	Tool website	GC%	List		Procedural	Perl	✓
PhytoCRISP-Ex [41]	Tool website		Score, filter		Procedural	Perl/bash	
CRISPOR [42]	Tool website	GC%	Score, list	Score	Procedural	Python	
CRISPR-DO [43]	BitBucket	PolyT, GC%, FA	Score	Score, filter	Procedural	Python	
sgRNAScorer2 [44]	Tool website			Score	ML	Python	
GuideScan [18]	BitBucket		Score, list		Procedural	Python	
FlashFry [19]	GitHub	PolyT, GC%, FA	Score, list	Score	Procedural	Java	
TUSCAN [27]	By request			Score	ML	Python	

The 18 selected tools analysed in this study; chronologically ordered by date of first publication. GC%: the tool calculates the guanine-cytosine content of the guide. FA: *feature aware* (i.e. accepts annotation). PolyT: the tool checks for poly-thymine sequences. SSE: the tool calculates the second structure and energy. *List*: the number of off-target sites is listed. *Filter*: the tool removes guides based on this rule. *Score*: the tool provides a numerical value for this rule. *ML*: machine learning. *Bulge support* indicates both DNA and RNA bulges.

Python (n = 11) and Perl (n = 5) are the most common programming languages, with CT-Finder and CRISPOR also implementing web-based interfaces via PHP (complemented by JavaScript, CSS, HTML, etc.). To improve run time, mm10db implements some of its components in C. SSC and FlashFry are implemented in C and Java, respectively. PhytoCRISP-Ex is a Perl-implemented tool, however, extensively utilises Linux bash commands for pre-processing. Configuration of tools is most commonly achieved via command-flags, however, tools such as Cas-Designer and CasFinder are configured via a text file. CHOPCHOP is configured via global variables in the main script file. Only Cas-Designer leveraged the GPU for additional computing resources. We ran this tool in both CPU and GPU modes when considering the computational performance.

SciPy [10] (inc. Numpy) and BioPython [11] were common packages utilised by the Python-based tools. CHOPCHOP and WU-CRISPR use SVMlight [12] and LibSVM [13], respectively; both being C implementations of support vector machines (SVM). Similarly, sgRNAScorer2 and TUSCAN utilise the machine learning modules from the SciKit-learn package [14]. The authors of sgRNAScorer2 supplied models for the 293T cell line and the hg19 and mm10 genomes. CHOPCHOP, GT-Scan, CRISPR-ERA, CCTop and CasFinder use Bowtie [15] for off-targeting; similarly to mm10db and CT-Finder with Bowtie2 [16]; while CRISPOR and CRISPR-DO utilise the Burrow-Wheelers Algorithm (BWA) [17]. GuideScan does not depend on external tools for off-targeting, and instead implements a trie structure for designing guides with greater specificity [18]. FlashFry benefits from its guide-to-genome aggregation method which is able to identify off-target sites in a single pass of its database. This achieved greater performance for FlashFry in comparison to BWA, as the number of mismatches and number of candidate guides increases [19]. Interestingly, [20] identified that Bowtie2 lacks the ability to rapidly identify all off-target sites with greater than two mismatches and that Cas-OFFinder [21] is a more suitable solution, however is more time expensive.

CHOPCHOP, Cas-Designer, mm10db, CCTop and CRISPR-DO provide ways to specify annotation files. For each of these tools, we provided the appropriate annotation in each test. CHOPCHOP utilises the annotations to indicate which gene or exon a candidate guide targets, and to allow the user to restrict the search region to particular genes. Cas-Designer utilises a custom-format annotation file, which describes the start and end positions of each exon on a given genome. This is used for designing candidate guides which specifically target exon regions. mm10db requires an annotation file in the UCSC RefGene format in order to generate a file containing sequences for all exons. CCTop utilises annotations for evaluating guides based on their distance to the closest exon, and for passing results to the UCSC genome browser as a custom track. None of the selected tools are restricted to particular genomes; all have been developed to allow for genomes of any organism to be provided. This includes personal genomes, for instance if applying these tools in contexts such as personalised medicine. DNA or RNA bulges were supported by two tools only, as described in Table 1. Genetic variation is not supported by any of the tools which we have included.

Some biological rules are shared across tools, such as: avoiding poly-thymine sequences [22], rejecting guides with inappropriate GC-content [23], counting and possibly considering the position of single-nucleotide polymorphisms (SNPs), and considering the secondary structure of the guides. Most tools report all targets that have been identified (sometimes with a score for specificity and/or efficiency) and rely on the user to determine whether a guide is appropriate for use, while mm10db actively filters guides and only reports ‘accepted’ ones (but ‘rejected’ targets, and reason for rejection, are still available in a separate file). WU-CRISPR and sgRNAScorer2 do not implement any of these rules through procedural-styled programming, but instead utilise machine learning models trained from experimental data. Furthermore, due to the age of some tools and the rapid growth of CRISPR-related research, the specifics of these procedural rules vary. For example, early studies describe the 10-12 base pairs adjacent to the PAM (the *seed* region) are more significant than the remainder of the guide [1, 24], but recent research contradicts this and suggests that one-to-five base pairs proximal to the PAM are more likely to be of significance [25]. Recent research has shown that specific motifs within the seed region reduce gene knock-out frequencies by up to 10-fold when present [26]. When evaluating tools, these may be factors that researchers would want to consider.

## Results

### Output

A similar set of candidate guides was generated by each tool, however, the start and end positions of identical guides often differed by up to 4 positions. This was seen due to some tools truncating the PAM sequence, or where zero-based positioning was used. An ellipsis was concatenated during normalisation for those that truncated it. All guides were aligned with one-based positioning as per the UCSC datasets, however, it is noted that Ensembl and Bowtie adhere to a zero-based system, explaining its usage in some tools. Seventeen of the tools produced output using the comma- or tab-separated values (CSV, TSV, respectively) formats, which makes their results easy to exploit for any post-processing. Five of the tools (GuideScan, CRISPR-ERA, mm10db, GT-Scan, CCTop and SSC) did not provide a header line to indicate the meaning of each column. GT-Scan differed as it produced an SQLite database containing a table of all candidate guides. Tests which were terminated did not produce output as the write-to-file routines had not been reached prior to termination.

### Output consensus

Here, we discuss the consensus between tools for the datasets derived from chr19. We focus on the ‘500k’ dataset, as this was the only dataset where each tool successfully completed a test. Similar results were observed on the larger datasets, for the tools that still managed to produce an output.

We define the consensus *C*_*i*,*j*_ between tool *i* and tool *j* as the proportion of guides produced by *i* that are also produced by *j*. Note that the value is not symmetric: while the intersection between the two set of guides is unchanged, the denominator for *C*_*i*,*j*_ and *C*_*j*,*i*_ is the number of guides produced by *i* and by *j*, respectively.

There is no ideal, absolute value for the consensus level. If two tools do not filter targets and simply report all candidate sites, one would expect a perfect consensus. This says nothing about the quality of the targets, but confirms that the tools behave as expected. On the other hand, for tools that actively filter targets, it is reasonable to expect a lower consensus level, since they use different criteria to select targets. The consensus level is a first layer of analysis, which needs to be complemented by experimental data.

**Fig 1:**
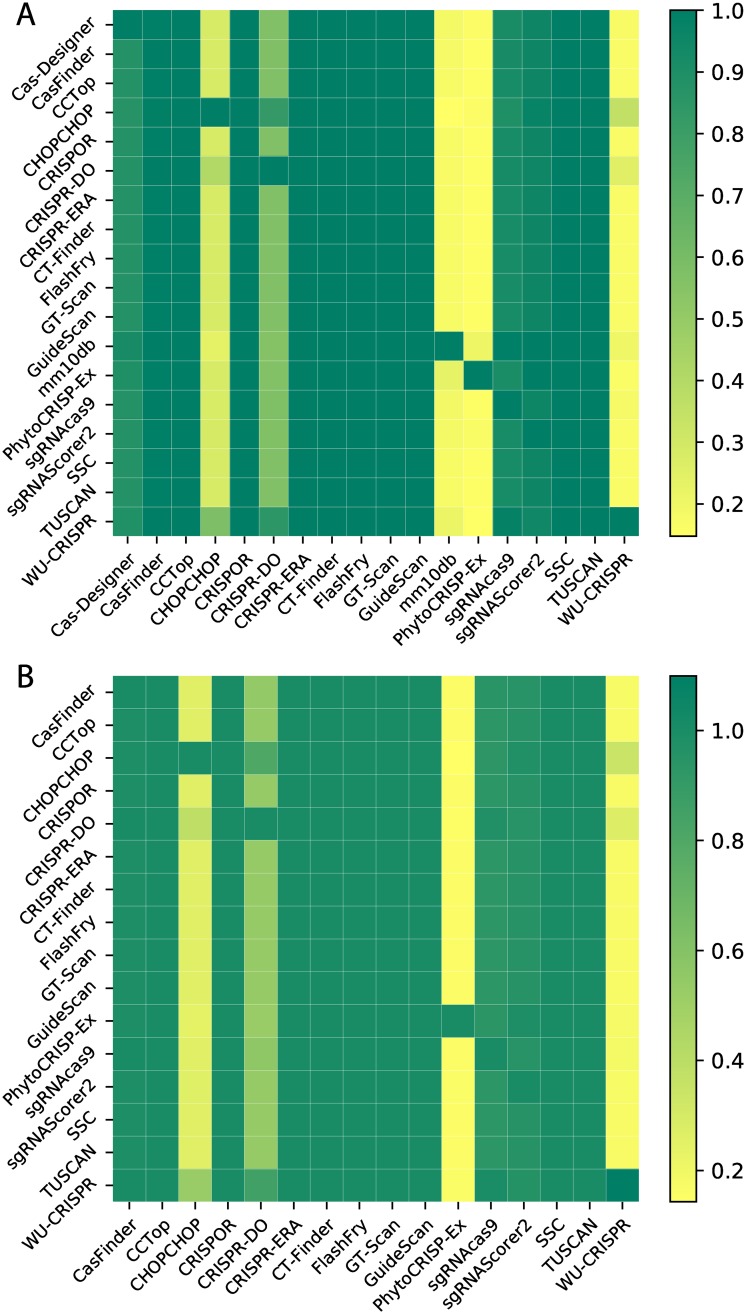
Consensus matrix (‘500k’ dataset). (A) Consensus between tools that target the entire input dataset. (B) Consensus between all tools, for coding regions only.

The consensus matrix *C* is shown in Fig 1A, and highlights that most tools do not filter targets. They report all possible targets, sometimes with a score. This leads to a high consensus between methods, typically 95% or more. The CHOPCHOP method, on the other hand, checks that a guanine is present at the twentieth nucleotide. If this is enforced, it reports only about a quarter of the targets that other tools produce. CRISPR-DO removes those that contain poly-thymine sequences and those with undesirable off-target effects. PhytoCRISP-Ex only considers as potential targets the guides that satisfy two rules: (i) they have at most two off-target sites with only one mistmatch anywhere else in the input genome, and (ii) their seed region (last 15 bases including the PAM sequence) is unique. These potential guides are then checked for the presence of restriction sites for pre-selected, common restriction enzymes. The consensus of WU-CRISPR with other tools was a maximum of 34.6%. This was due to the tool applying pre-filters to eliminate guides before being evaluated by the SVM model. Some of the pre-filters included: folding and binding properties as well as position specific guide contents. Following this, the tool only reports guides which match the model.

Five tools (CHOPCHOP, Cas-Designer, mm10db, CCTop and CRISPR-DO) focus, or give the option to focus, on identifying targets located on exons. Fig 1B shows the consensus matrix for these regions, across all tools. As before, there is a high consensus between tools that do not reject targets. For tools that more strictly select which targets to report (CHOPCHOP and mm10db), the consensus is again around 15-30%. Interestingly, the consensus between CHOPCHOP and mm10db is low. This highlights that tools are using different selection criteria, and this leads to the identification of distinct targets.

Only one tool (mm10db) provides a detailed report identifying reasons for rejection. These reasons can be used to analyse the output of the other tools and gain some insight into their behaviour. It is worth noting that mm10db applies filters sequentially. For instance, a guide rejected for multiple exact matches in the genome might have also been rejected for its GC content if it had made it to that filter. The results are still informative. The main reasons were high GC-content (approx. 62%), a poor secondary structure or energy (19%), and multiple exact matches (16%). Off-target scoring is the last step so, despite being strict, it necessarily contributes to a small fraction of the rejections. Here, this is made even smaller (< 0.05%) because the small input size limits the risk of having a large number of similar off-target sites.

**Fig 2:**
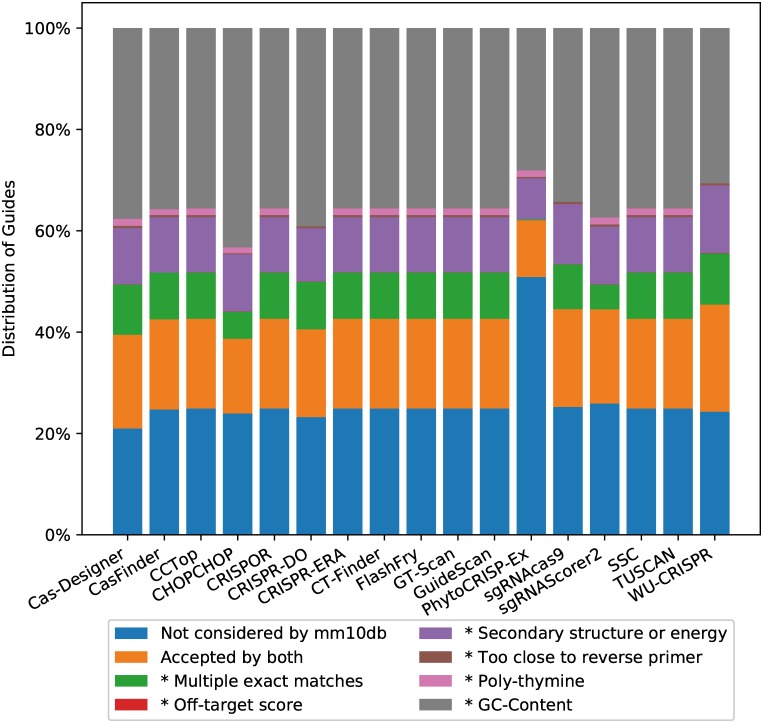
Reasons for rejection. This plot shows, for each tool, the proportion of accepted guides that have been rejected by mm10db, and the reason for doing so. An asterisk (*) indicates the category relates to a rule implemented in mm10db for rejecting guides. mm10db is used as a reference point because it provides a reason for any guide it rejects.

From Fig 1, we know that up to 83% of guides proposed by other tools are rejected by mm10db. For each tool, we also explored the rejection reasons given by mm10db. This is summarised in Fig 2 for the ‘500k’ dataset. Rejection due to GC content is the most significant reason, followed by secondary structure or energy.

Importantly, a number of guides (on average around 10%) proposed by these tools are rejected by mm10db due to multiple exact matches in the genome. For instance, guide CTCCTGAGTGCTGGGATTAAAGG is reported by 10 tools when analysing the ‘500k’ dataset, which contains the guide at five loci as perfect matches. This guide targets two distinct genes on chr19 alone: Prpf19 at chr19:10,907,131-10,907,153 and Gm10143 at chr19:10,201,455-10,201,477. Ideally, this guide would not appear in the output from a tool due to its lack of specificity: there is no practical application where such a guide would be useful. Furthermore, none of the tools that deal with specificity by reporting the number of sites with *k* mismatches (*k* ≤ 5) actually reported the correct number of off-targets for this guide. Cas-Designer and GT-Scan reported zero perfect matches. CHOPCHOP reported 50 perfect matches (Bowtie was used by CHOPCHOP for this task, however, was limited to report 50 alignments). Aligning this guide on the full chr19 using Bowtie (*-v 0 -a*), we found 241 perfect matches. PhytoCRISP-Ex did not report any guides that we later identified as having multiple matches in the genome. This is because, as mm10db, it is strict about filtering out guides that may have off-target partial matches.

Fig 2 also shows that, for tools that report all guides, roughly a quarter of their output has not been considered (i.e. neither accepted nor rejected) by mm10db. This is due to the regular expression it is using to extract candidates ([*ACG*][*ACGT*]^20^*GG*), which excludes candidates starting with a T, while other tools do not.

### Experimental dataset

We then considered the performance of tools that either filter targets or provide a score which predicts efficiency. We created two artificial genomes, each containing guide sequences for which experimental data on their efficiency is available (see Data Preparation section). We refer to these artificial genomes based on their originating datasets: *Doench* and *Xu*. Note that here, we were only focusing on assessing whether efficiency is correctly predicted. Using an artificial input was therefore crucial. If we were scoring the guides against the whole genome, some could be rejected due to other factors (e.g. off-target considerations) that are not related to their predicted efficiency. A guide rejected because of the off-target risk would tells us nothing about the ability of the tool to predict whether the guide is efficient. By using an artificial genome, we can ensure that each guide has absolutely no off-target risk, and can therefore focus on the efficiency prediction. In this context, a perfect tool would accept all the guides experimentally shown to be efficient, and would reject all the inefficient ones.

**Fig 3:**
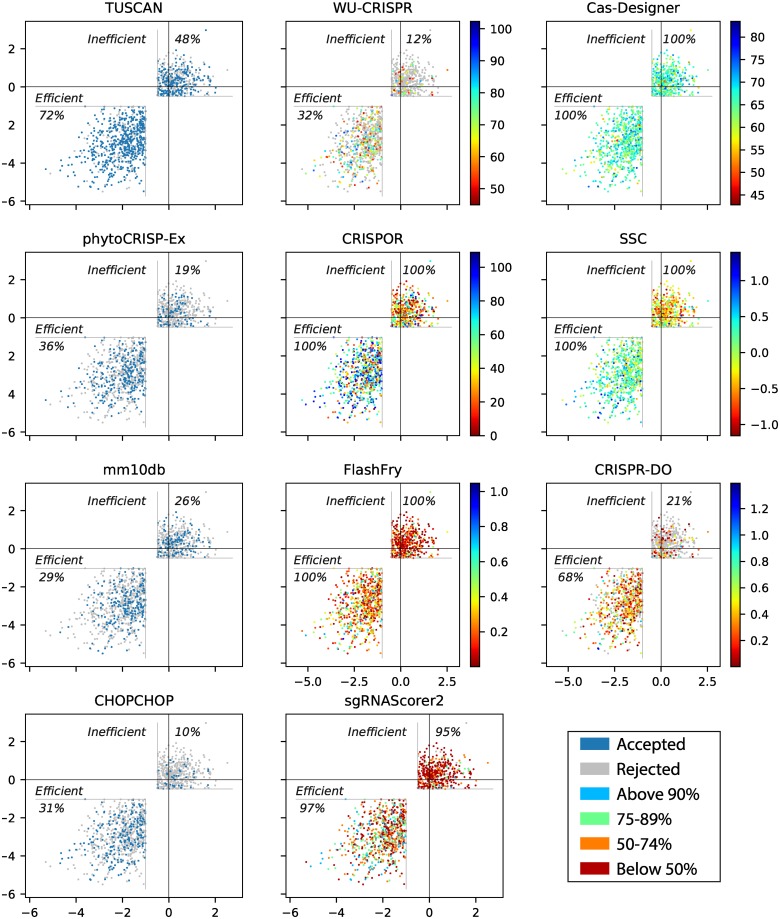
Tool performance on Xu guides. For TUSCAN, mm10db, CHOPCHOP and phytoCRISP-Ex, colours indicate the whether a guide was accepted by the tool. For sgRNAScorer2, each colour indicates which percentile a particular guide scored in. For all others, colours represent the score distribution over the dataset.

**Fig 4:**
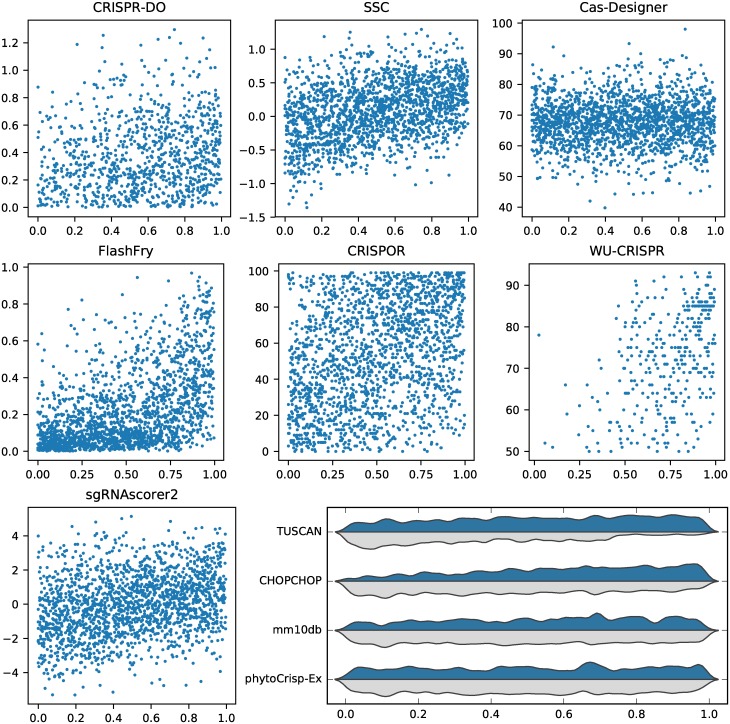
Tool performance on Doench guides. For tools that score guides, the experimental gene rank score versus score reported by tool is plotted. For TUSCAN, mm10db, CHOPCHOP and phytoCRISP-Ex, the distribution of accepted and rejected guides is shown, based on their associated gene rank score.

The results are shown in Figs 3 and 4. In our analysis we considered the precision of the tools, which is the proportion of guides predicted to be efficient that actually were experimentally found to be efficient.

For the Xu dataset, CHOPCHOP accepted 273 guides, 84.3% of which were ‘efficient’, and mm10db accepted 330, 65.2% of which were ‘efficient’. Incorporating CHOPCHOP’s rule of having a G at position 20 into mm10db would bring its proportion of ‘efficient’ guides to 84.4%. The results also highlight the value in considering multiple selection approaches: only 25.1% of the ‘efficient’ guides identified by mm10db have also been selected CHOPCHOP.

The sgRNAScorer2 user manual states that scores should be used in a *relative* manner as opposed to *absolute*: the higher the score, the better the predicted activity, but there is no threshold to classify between predicted efficiency and inefficiency. The 50th, 75th and 90th percentiles used in Fig 3 are therefore for visualisation only. Instead, we extracted every pair (*a*,*b*) of guides where *a* was experimentally considered ‘efficient’ and *b* ‘inefficient’, and checked whether *a* had a higher predicted activity than *b*. This was true for 76.8% of them.

CRISPR-DO and WU-CRISPR did not report all the guides provided in the artificial genome. For the guides where a score is available, the tools had a precision of 87.3% and 81.8%, respectively.

The guides which FlashFry scored higher mostly appear in the efficient region in Fig 3. If we use the recommended threshold for the tool, 84.4% of the selected guides are efficient. Similarly, CRISPOR (which reports the Azimuth score [20]) has a precision of 77.4%, and SSC 85.1%. Overall, the result for these tools suggests that they are able to score guides with some accuracy.

We used the classification model for TUSCAN, which is reported to outperform sgRNAScorer2 and WU-CRISPR [27]. Here, we found 71.5% of the guides that TUSCAN accepted were experimentally shown to be efficient. Among the tools that filter targets (rather than scoring them), TUSCAN had the highest recall (i.e. proportion of efficient guides that are selected).

CRISPR-DO had the highest precision across all tools (87.3%), whilst Cas-Designer had the lowest (61.2%).

For the eleven tools considered, there is a very low overall consensus; only one guide in the entire dataset was selected by all of the tools.

For the Doench dataset, guides from nine transcripts were provided and only guides in the top 20% for each transcript were considered as efficient [28]. For this dataset, we found that the precision for each tool was significantly lower compared to Xu. This was expected because of the high threshold used to define efficiency in this study. Only two tools have a precision above 50%: WU-CRISPR, which was trained on this dataset, and FlashFry, which is also using a scoring method originally derived from this dataset. TUSCAN was partially trained on this dataset but has a precision of 24.5%.

All the other tools have a precision between 20.2% (Cas-Designer) and 30.4% (CRISPR-DO), as summarised in Table 2. Only two tools (mm10db and CHOPCHOP) have an accuracy above 65%.

**Table 2:** Precision of tools on experimental data.

Tool name	Doench	Xu
**CRISPR-DO**	30.40%	87.28%
**Cas-Designer**	20.18%	61.17%
**mm10db**	22.66%	65.15%
**CHOPCHOP**	29.39%	84.25%
**WU-CRISPR**	50.82%	81.82%
**sgRNAScorer2**	27.12%	83.26%
**PhytoCRISP-Ex**	23.38%	76.44%
**CRISPOR**	26.41%	77.40%
**FlashFry**	58.85%	84.40%
**TUSCAN**	24.51%	71.51%
**SSC**	27.53%	85.13%

The precision, which is the proportion of guides predicted to be efficient that actually were experimentally found to be efficient, of each tool per dataset.

Fig 4 shows the performance of each tool on this dataset. For tools which provide a score, we show their prediction as a function of the gene rank percentage. For FlashFry, guides with low gene rank percentages are frequently scored low. WU-CRISPR reports high scores for many guides with high gene rank percentages. TUSCAN accepts a large majority of efficient guides. For SSC and sgRNAScorer2, there is a very weak trend for higher scores as the gene rank is increasing. The Azimuth score (as reported by CRISPOR), CRISPR-DO and Cas-Designer are not showing any clear trend. For tools which accept or reject guides, we show the distribution of these two categories over the gene rank percentage. For mm10db higher ranked guides are more likely to be accepted, and lower ranked are more likely to be rejected. For CHOPCHOP, this is not as pronounced and for phytoCRISP-Ex there is no trend.

Again there is a very low overall consensus; only three guides in the entire dataset were selected by all eleven tools.

### Computational performance analysis

Even though it is often overlooked when methods are initially presented, their computational performance is an important consideration. There are a number of applications of CRISPR where it can be crucial: large input genomes, time constraints to obtain the results, large number of non-reference genomes to analyse, etc.

In terms of time requirements, our hypothesis was that any efficient scoring or filtering would scale linearly with the number of targets to process (since they are individually assessed), and therefore almost linearly with the input genome size. However, for the specificity analysis, each guide needs to be assessed against any other candidates, which could result in quadratic growth. Another possible limitation is the memory requirement of the tools.

**Fig 5:**
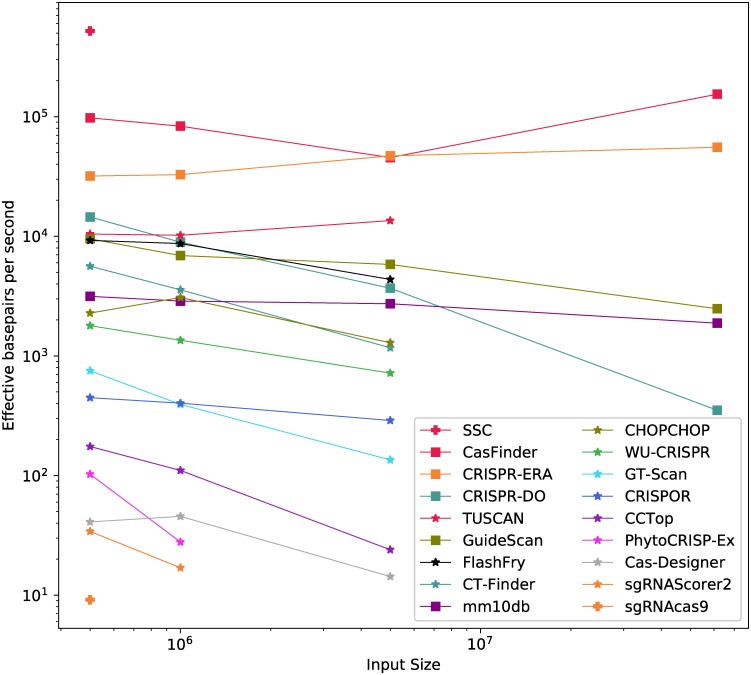
Normalised processing speed. Measured in effective basepairs per second; taking into account the total length of the sequences considered by any given tool. Three performance groups have been identified, and are shown with different symbols for their markers.

Our results are shown in Table 3 and Fig 5. Only five tools successfully completed the four tests: CasFinder, CRISPR-ERA, mm10db, GuideScan and CRISPR-DO. GuideScan just reports all possible targets, providing no scoring on predicted efficiency, and was therefore expected to be fast. CasFinder and CRISPR-ERA provide a score, but it was identical across the dataset and was therefore not informative. On the other hand, mm10db runs candidate guides through a number of filters and provides a meaningful score for the off-target risk, so its speed is worth noting.

**Table 3:** Run time per test.

	500k	1m	5m	Full chr19
**CasFinder**	00:00:05	00:00:12	00:01:50	00:06:38
**CRISPR-ERA**	00:00:16	00:00:31	00:01:46	00:18:27
**mm10db**	00:00:17	00:00:49	00:01:53	00:26:03
**CRISPR-DO**	00:00:34	00:01:52	00:22:37	00:32:36
**GuideScan**	00:00:52	00:02:25	00:14:19	06:53:07
**TUSCAN**	00:00:48	00:01:38	00:06:10	#
**FlashFry**	00:00:54	00:01:54	00:19:07	*
**CT-Finder**	00:01:29	00:04:41	01:11:12	*
**GT-Scan**	00:11:05	00:42:24	10:16:00	#
**Cas-Designer CPU**	00:21:21	00:51:12	06:00:30	*
**Cas-Designer GPU**	00:10:06	00:38:57	04:40:47	*
**CCTop**	00:47:39	02:31:00	09:57:40	*
**CHOPCHOP**	00:00:23	00:00:45	00:04:00	#
**CRISPOR**	00:18:37	00:41:22	04:48:39	#
**WU-CRISPR**	00:04:39	00:12:20	01:55:57	^
**PhytoCRISP-Ex**	01:21:13	09:59:18	*	-
**sgRNAScorer2**	04:04:06	16:23:31	*	-
**sgRNAcas9**	15:11:46	*	-	-
**SSC**	00:00:01	^	-	-

Run time denoted as hh:mm:ss for each tool and dataset size. Some tools exceeded the 72-hour limit (*), or were terminated by the the operating system’s out-of-memory killer (#). Any tool failing a test was not considered for larger input sizes (-). WU-CRISPR failed on ‘full’ due to the tool rejecting inputs containing *N*, and SSC could not process inputs larger than 500,000 bases in length (^).

CHOPCHOP saturated both physical memory and allocated swap space (as monitored by SBS), resulting in the out-of-memory killer (OOMK) terminating its run on the full chromosome 19 (approx. 2% of the mouse genome). sgRNAScorer2 was extremely slow, taking more 4 hours to process the 500k dataset (when the other tools took between 5 seconds and 21 minutes). It did not manage to process the 5m dataset in less than 3 days. FlashFry was performing well in the tests below ‘full’, however, the Java Virtual Machine was exhausted of memory when completing the final test.

Four other tools failed to satisfy the time constraint, all on the full chromosome 19 (Cas-Designer, CT-Finder, GT-Scan and CCTop). CRISPOR, GT-Scan and TUSCAN had memory problems similar to those of CHOPCHOP and were terminated on that same dataset.

As part of our analysis we also considered the use of multi-threading. CHOPCHOP, GuideScan, mm10db, FlashFry and sgRNAScorer2 are the only tools which have implemented multi-threaded routines. CHOPCHOP and GuideScan do not allow the user to specify the number of threads to spawn, but instead, spawn threads according to the number of CPU cores. mm10db provides command-flags to specify the number of threads for itself and Bowtie; we specified 128 and 8 threads, respectively. Cas-Finder, CT-Finder and CHOPCHOP utilise Bowtie in single-threaded mode.

We ran Cas-Designer in GPU mode and found this provided a performance boost. However, this was still not enough to allow the full dataset to be processed within the time limit. Ideally, future tools (or future versions of the tools benchmarked here) should also leverage GPU resources.

SBS monitored the physical memory and allocated swap space usage. CRISPOR, CHOPCHOP, GT-Scan and TUSCAN saturated both of these memory spaces, resulting in the out-of-memory killer (OOMK) terminating each tool on the full dataset test. Cas-Designer saturated the swap space, however did not trigger the OOMK in the same test.

## Discussion

A large number of tools have been proposed to assist in the design of CRISPR-Cas9 guides. Many of them, three from our study in particular, have been successfully used experimentally: CasFinder has been used for designing guides from the human genome to target specific genes [29], CRISPR-ERA has been used for designing guides to target the HIV-1 viral genome [30] and mm10db has been used for whole-genome analysis of the mouse genome [6].

However, there has been little comparison of their performance. This paper address this gap by benchmarking 18 tools in terms of their computational behaviour as well as the guides they produce. Our results show that only five tools (CasFinder, CRISPR-ERA, CRISPR-DO, GuideScan and mm10db) can claim to analyse large inputs rapidly and would be readily available to process entire genomes, especially larger ones.

Many tools are not selective in designing guides: they report all candidates, and therefore have a high consensus between them. On the other hand, eleven tools provide clear predictions of guide efficiency, as listed in Table 2. We assessed their performance on two collections of experimentally validated guides. All eleven tools produced a majority of efficient guides for the Xu dataset but much fewer for the Doench dataset. For both datasets, there was limited overlap between tools. This has shown that the tools do have the ability to detect some efficient guides, but also that they are not completely precise and still have low recall. Improved methods are needed.

Taken together, the results suggest that mm10db has currently the best balance between speed and filtering/scoring of the guides for whole-genome analysis. When working on a small number of genes, the much-slower but more predictive CRISPR-DO can be preferred. CHOPCHOP had issues scaling to the full dataset, but would otherwise be a good choice as well. Importantly, the results also emphasise the need for further refinement of CRISPR-Cas9 guide design tools. This benchmark provides a clear direction for future work on optimising computational performance and combining multiple design approaches.

## Materials and methods

Given the range of rules and implementations, it is crucial to benchmark guide design tools and compare their performance. In this section, we describe how each tool is evaluated based on compute requirements, features and output.

### Data preparation

The initial data from our benchmarking is based on the *GRCm38/mm10* mouse genome assembly, available via the University of California, Santa Cruz (UCSC). We downloaded chr19, and extracted three datasets of increasing length: 500k, 1m, and 5m nucleotides, all starting from position 10,000,000. These datasets, and the whole chromosome, are used for testing.

For each of these four configurations, we created all the files required by any of the tools: custom annotation file (derived from the *refGene* table available via UCSC), 2bit compression file, Bowtie and Bowtie2 indexes, and Burrows-Wheeler Aligner file.

To complement these datasets, we have also used two collections of guides for which experimental data is available [28, 31]. One collection, *Xu*, contains 1169 guides which were used in a screening experiment, with 731 deemed to be ‘efficient’ based on analysis of the gene knock-outs. The other, *Doench*, contains 371 ‘efficient’ and 1470 ‘inefficient’ guides. Knowing the experimental quality of a guide can give further insight into the quality of computational techniques for evaluating guides.

We constructed two artificial sequences that contain the guides from each dataset, interspaced by 50 N’s to ensure that unexpected overlapping targets cannot be detected. As before, we generated all the supporting files required for this input.

The tools were not optimised for a specific organism, and the choice of mm10 for the initial tests, or of data from human cell lines for the experimental validation, should not impact the results.

Our datasets are available in S1 Dataset.

### Performance benchmarking

Our *Software Benchmarking Script* (SBS) tool is implemented in Python 2.7, and uses the Processes and System Utilisation (PSUtil, version >= 5.4.4) module for process-specific monitoring of system resources. When launched, the user is required to pass a bash command and an output directory via command-line flags. The audit routine begins after the bash command is executed. The parent process and all descendants are monitored at each polling event (PE).

The current wall-time, CPU and memory usage, disk interaction (DIO), number of threads and number of children are recorded. Wall-time was preferred to CPU-time as it is the human-perceived completion time. SBS reports the instantaneous resident set size (RSS) usage and virtual memory usage. DIO includes both the number and size of read/write operations. At each PE, an aggregate of the parent and child data is calculated and written to file. Additionally, the bash commands which launched each child process are logged.

The PE routine continues until the parent process ends or 72 hours is exceeded. This limit was imposed as we aimed to discuss tools with potential for whole-genome analysis, and those that cannot analyse chr19 (2.25% of mm10 total size) within this limit are deemed inappropriate for the task.

All tests were performed on a Linux workstation with Intel Core i7-5960X (3.0 GHz), 32 GB RAM, 32 GB allocated swap space, and Samsung PM87 SSD. We used Python v2.7 and Perl v5.22.1. This machine exceeds the specifications of some workstations, however, it is expected that a user would require a similar machine or better in order to achieve whole-genome analysis.

SBS is available on GitHub at https://github.com/jakeb1996/SBS.

### Output normalisation and comparison

Each tool had its own output format, so we normalised the results as: tool name, candidate guide sequence, start position, end position and strand (written to file in the CSV format). During this process, the start and end values were aligned with one-based positioning, as per the UCSC datasets. An ellipsis was concatenated to guides which were lacking a PAM sequence.

To determine which tools shared common guides, a script aggregated all non-duplicate guides and recorded which tool produced subsequent occurrences of the guide. A guide was considered a duplicate with a previously observed guide when the 3’ positions were equal and when they were reported to target the same DNA strand. A separate script analysed each normalised guide to determine whether it targeted a gene coding region (based on the UCSC annotation data).

## Supporting information

S1 Dataset Mouse and experimental datasets. Tab-separated values (TSV) files containing a list of exons for the 500k, 1m, 5m and full datasets. Comma-separated values (CSV) files containing the accepted and rejected guides as reported by mm10db in the 500k test. The two experimentally validated guides datasets, which contain flags indicating whether a guide was experimentally efficient or inefficient. Scripts used to generated the artificial genomes.
(ZIP)

S1 Protocol Data analysis and plotting scripts. The scripts used to generate the plots in this paper. The scripts used to normalise the output generated by each test and to extract those guides which target coding regions.
(ZIP)

S2 Protocol Tool setup and run commands. A RMarkdown file and knitted HTML edition of this file, which describes: the installation process of a tool, any modifications made to the source code for a tool, and a sample command which was used to run a test for each tool.
(ZIP)
