# Supplementary material for: A benchmark of computational CRISPR-Cas9 guide design methods
Source: PLoS Comput Biol. 2019 Aug 29;15(8):e1007274. doi: 10.1371/journal.pcbi.1007274 (PMC6738662; doi:10.1371/journal.pcbi.1007274)
Supplement: S2 Protocol — A RMarkdown file and knitted HTML edition of this file, which describes: the installation process of a tool, any modifications made to the source code for a tool, and a sample command which was used to run a test for each tool. (ZIP) [file pcbi.1007274.s003.zip › S3_Protocol/instructionsR.html]

# CRISPR-Cas9 Guide Design Methods - Setup Instructions

**A Benchmark of Computational CRISPR-Cas9 Guide Design Methods**

Jacob Bradford, Dimitri Perrin. 2019.

---

### Preamble

- This document describes for following information for each of the CRISPR-Cas9 guide design methods which we analysed:

  - Setup instructions
  - Code modifications
  - Installed dependencies and versions
  - Method version number or commit hash
  - Run commands via our benchmarker *Software Benchmarking Script* (SBS)
- We have provided the instructions for running each tool against the `500k` dataset, however, this can easily be modified for the datasets of larger size. If not documented otherwise, changing the datset supplied to the tool requires changing `500k` to `1m`, `5m`, or `full`.
- For those tools available through an online git service, we have provided the date and hash of the commit which was pulled to our local machine.
- For those tools not in a version control system, we have provided the date for when we obtained the source code.
- If a Python virtualenv was not required for a tool, or was not set up for a tool, then a virtualenv with the following configuration was used:

```
Package       Version
------------- -------
biopython     1.73
numpy         1.16.0
pip           10.0.1
pkg-resources 0.0.0
psutil        5.4.6
setuptools    39.2.0
wheel         0.31.1
```

(To obtain Python package versions, we ran `pip list`).

---

## CasFinder

**Version:** 5 December 2014

**Tool specific virtualenv:** No

**Setup:**

Update `CASFINDER_CONFIG.txt`:

```
#### EXECUTABLES
bowtie_executable   /home/jake/bowtie-1.2.2/bowtie
casvalue_program    /home/jake/tools/CasFinder/CasValue_v2.pl
#### GENOMES
genome  mm10
bowtie_index    /home/jake/genomes/mm10-ucsc-mod/chr19/500k/chr19.fa
chromosomes /home/jake/genomes/mm10-ucsc-mod/chr19/500k/
...
#chr12  chr12.fa
#chr13  chr13.fa
#chr14  chr14.fa
#chr15  chr15.fa
#chr16  chr16.fa
#chr17  chr17.fa
#chr18  chr18.fa
chr19   chr19.fa
#chr20  chr20.fa
#chr21  chr21.fa
#chr22  chr22.fa
#chrX   chrX.fa
#chrY   chrY.fa
end chromosomes
```

**Run:**

```
python /home/jake/sbs/sbs.py -c "
perl /home/jake/tools/CasFinder/CasFinder.pl 
-i /home/jake/genomes/mm10-ucsc-mod/chr19/500k/chr19.fa 
-o /home/jake/tools/CasFinder/output/0006
" -o /home/jake/sbs/output/CasFinder-0006/CasFinder-0006 -l y -s 0.1
```

---

## CHOPCHOP

**Version**: 26 September 2017 (`384743ca145db650e4658923d3f865dd0f7b337a`)

**Tool specific virtualenv:** Yes

**virtualenv packages:**

```
Package         Version
--------------- -------
biopython       1.71
numpy           1.14.3
pandas          0.23.0
pip             10.0.1
pkg-resources   0.0.0
psutil          5.4.5
python-dateutil 2.7.3
pytz            2018.4
scipy           1.1.0
setuptools      39.2.0
six             1.11.0
wheel           0.31.1
```

**Setup:**

CHOPCHOP required configuring via globally declared variables in `chopchop.py`:

```
# PATHs
PRIMER3 = "/home/jake/tools/chopchop/primer3_core"
BOWTIE = "/home/jake/bowtie-1.2.2/bowtie"
TWOBITTOFA = "/home/jake/tools/chopchop/twoBitToFa"
TWOBIT_INDEX_DIR = "/home/jake/genomes/ucsc/mm10/bigZips"
BOWTIE_INDEX_DIR = "/home/jake/genomes/mm10-ucsc-mod/chr19/500k"
ISOFORMS_INDEX_DIR = "/your/full/path/to/ebwt_transcriptome_folder" #only when using --isoforms
GENE_TABLE_INDEX_DIR = "/home/jake/genomes/ucsc/GRCm38-mm10"
```

**Run:**

```
mv /home/jake/tools/chopchop/chopchop.py /home/jake/tools/chopchop/chopchop-do-not-delete.py
mv /home/jake/tools/chopchop/chopchop-500k.py /home/jake/tools/chopchop/chopchop.py

python /home/jake/sbs/sbs.py -c "
python /home/jake/tools/chopchop/chopchop.py 
-G chr19.fa 
-o /home/jake/tools/chopchop/output-0019 
-F /home/jake/genomes/mm10-ucsc-mod/chr19/500k/chr19.fa
" -o /home/jake/sbs/output/chopchop-0019/chopchop-0019 -l y -s 1

mv /home/jake/tools/chopchop/chopchop.py /home/jake/tools/chopchop/chopchop-500k.py
mv /home/jake/tools/chopchop/chopchop-do-not-delete.py /home/jake/tools/chopchop/chopchop.py
```

---

## sgRNACas9

**Version:** 3.0.5

**Tool specific virtualenv:** Yes

**Code modifications:**

- Line 28 (from -> to):

  `my ($Inputfile_Fasta, $truncat, $GC_l, $GC_m, $Genome, $Option, $Type, $Seqmap_vesion, $Num_mismatch, $offset_s, $offset_e, $path);`

  `my ($Inputfile_Fasta, $truncat, $GC_l, $GC_m, $Genome, $Option, $Type, $Seqmap_vesion, $Num_mismatch, $offset_s, $offset_e, $path, $jakesTestNumber);`
- Line 41: Added another CLI parameter:

  `, (\n) "j=i" => \$testNumber, #jakes test number`
- Line 54 (added):

  `$jakesTestNumber ||= "0001";`
- Find and replace (from -> to):

  `$dir/sgRNAcas9.report_$truncat.$Option.$Inputfile_Fasta`

  `$dir/sgRNAcas9.report_$truncat.$Option.$jakesTestNumber`

**Run:**

```
python /home/jake/sbs/sbs.py -c "
perl /home/jake/tools/sgRNAcas9_3.0.5/sgRNAcas9_3.0.5.pl 
-i /home/jake/genomes/mm10-ucsc-mod/chr19/500k/chr19.fa 
-o b 
-p /home/jake/tools/sgRNAcas9_3.0.5/output 
-g /home/jake/genomes/mm10-ucsc-mod/chr19/500k/chr19.fa 
-t s 
-j 0002
" -o /home/jake/sbs/output/sgRNAcas9-0002/sgRNAcas9-0002 -l y -s 1
```

---

## GT-Scan

**Version:** 1.31

**Tool specific virtualenv:** No

**Code modifications:**

- Line 8 of `config.ini`

  `ref_genome_dir = /media/dperrin/Data2/jake/genomes/mm10-ucsc-mod/chr19/full`

**Run:**

```
python /home/jake/sbs/sbs.py -c "
python /home/jake/tools/gt-scan_1.3/gt-scan.py 
-f /home/jake/genomes/mm10-ucsc-mod/chr19/500k/chr19.fa 
-g /home/jake/genomes/mm10-ucsc-mod/chr19/500k/chr19.fa 
-n gt-scan1.31-0001 
-r xxxxxxxxxxxxXXXXXXXXNGG
" -o /home/jake/sbs/output/gt-scan1.31-0001/gt-scan1.31-0001 -l y -s 1
```

---

## CCTOP

**Version:** 27 January 2018 (`95ea199ba2b65963adecdc1a2bf555c9171bb622`)

**Tool specific virtualenv:** Yes

**virtualenv packages:**

```
Package       Version
------------- -------
bx-python     0.8.1
numpy         1.14.5
pip           10.0.1
pkg-resources 0.0.0
psutil        5.4.5
python-lzo    1.12
setuptools    39.1.0
six           1.11.0
wheel         0.31.1
```

**Setup:**

- Update `GRCm38-p6-mm10-chr19-exons.bed` & `GRCm38-p6-mm10-chr19-genes.bed` chromosome column (first column, tab separated) to match the header line of the `chr19.fa` used

  Example:

  ```
  $ head -n1 /home/jake/genomes/mm10-ucsc-mod/chr19/100k/chr19.fa:
  ">ucsc-mm10-chr19-500k-extract[10000000-10100000]"
  ```

  Therefore, `GRCm38-p6-mm10-chr19-exons.bed` & `GRCm38-p6-mm10-chr19-genes.bed`, first columns (tab-separated) should be: `ucsc-mm10-chr19-500k-extract[10000000-10100000]`

**Run:**

```
python sbs.py -c "
python /home/jake/tools/cctop_standalone/CCTop.py 
--input /home/jake/genomes/mm10-ucsc-mod/chr19/500k/chr19.fa 
--index /home/jake/genomes/mm10-ucsc-mod/chr19/500k/chr19.fa 
--bowtie /home/jake/bowtie-1.2.2 
--output /home/jake/tools/cctop_standalone/output/0017/ 
--exonsFile /home/jake/genomes/mm10-ucsc-mod/GRCm38-p6-mm10-chr19-exons.bed 
--genesFile /home/jake/genomes/mm10-ucsc-mod/GRCm38-p6-mm10-chr19-genes.bed
" -o /home/jake/sbs/output/cctop-0017/cctop-0017 -l y -s 1
```

---

## SSC

**Version:** 0.1

**Tool specific virtualenv:** No

**Code modifications:**

- Line 18 of `Faster2Spacer.c`:

  ```
  #define MAX_SEQ_LEN 10000
  #define MAX_SEQ_LEN 650000
  ```

To run SSC, we wrote a brief bash script to automate the pipepine, `0002.sh`:

```
#!/bin/bash
./bin/Fasta2Spacer -5 20 -3 10 -i /home/jake/genomes/mm10-ucsc-mod/chr19/500k/chr19.fa -o 0002.spcr
./bin/SSC -i 0002.spcr -o 0002.scor -m /home/jake/tools/SSC0.1/matrix/human_mouse_CRISPR_KO_30bp.matrix -l 30
```

**Run:**

```
cd /home/jake/tools/SSC0.1/

python /home/jake/sbs/sbs.py -c "./0002.sh" -o /home/jake/sbs/output/ssc-0002/ssc-0002 -l y -s 1
```

---

## CRISPR-ERA

**Date obtained:** 26 April 2018

**Tool specific virtualenv:** No

**Run:**

```
python /home/jake/sbs/sbs.py -c "
perl /home/jake/tools/CRISPR-ERA/find_all_sgRNA_z_f_c_y.pl /home/jake/genomes/mm10-ucsc-mod/chr19/500k/chr19.fa output/0002-out_sgRNA.txt output/0002-out_sgRNA_fasta.txt output/0002-out_sgRNA_gc_t.txt output/0002-out_nag_fasta.txt output/0002-out_no_sgRNA.txt
" -o /home/jake/sbs/output/CRISPR-ERA-0002/CRISPR-ERA-0002 -l y -s 0.1
```

---

## WU-CRISPR

**Version:** 15 September 2015 (`710716651741109f77677cd25c9cd2904fd28407`)

**Tool specific virtualenv:** No

**Code modifications:**

- Line 121 in `wu-crispr.pl` (from -> to):

  `print "Error: Sequence is longer than 100,000 bases. \n\tWU-CRISPR will now now proceed to the next sequence.\n\n" and next if length ($submittedSeq)>100000;`

  `print "Error: Sequence is longer than 10,000,000,000,000 bases. \n\tWU-CRISPR will now now proceed to the next sequence.\n\n" and next if length ($submittedSeq)>10000000000000;`

**Run:**

```
python /home/jake/sbs/sbs.py -c "
perl wu-crispr.pl 
-f /home/jake/genomes/mm10-ucsc-mod/chr19/500k/chr19.fa
" -o /home/jake/sbs/output/wu-crispr-0002/wu-crispr-0002 -l y -s 1
```

---

## Cas-Designer

**Date obtained:** 8 May 2018

**Tool specific virtualenv:** Yes

**virtualenv packages:**

```
Package       Version
------------- -------
pip           10.0.1
pkg-resources 0.0.0
psutil        5.4.5
setuptools    39.1.0
wheel         0.31.0
```

**Code modifications:**

- Line 295 in `cas-designer.py` (from -> to):

  `p = Popen( ('cas-offinder-bulge', f.name, 'G', f.name + "_out"), stdout=PIPE, stderr=PIPE )`

  `p = Popen( ('cas-offinder-bulge', f.name, 'C', f.name + "_out"), stdout=PIPE, stderr=PIPE )`

Configuration file (`config-0022`):

```
/media/dperrin/Data2/jake/genomes/mm10-ucsc-mod/chr19/500k
/media/dperrin/Data2/jake/genomes/mm10-ucsc-mod/chr19/500k/chr19.fa
20
NGG
NRG
5
2
2
/media/dperrin/Data2/jake/genomes/mm10-ucsc-mod/exon-regions-for-cas-designer/refGene-chr19-10m-500k-adjusted.csv.cd
```

**Run:**

```
python sbs.py -c "
python /home/jake/tools/cas-designer/cas-designer.py /home/jake/tools/cas-designer/config-0022
" -l y -o /home/jake/sbs/output/cas-designer-0022/cas-designer-0022 -s 1
```

---

## mm10db

**Version:** 1 April 2018\* (`92d208c8dd556e68acdb33c978c2ba4c077377ed`)

\* obtained from authors prior to release as GitHub repository

**Setup:**

1. Setup the mm10\_input directory (hard coded)

   ```
   ln -s /home/jake/genomes/mm10-ucsc-mod/chr19/500k/ /home/jake/tools/mm10-CRISPR-DB/mm10_input
   ```
2. Prepare gene lists. This will generate file(s) in the `mm10_input` directory.

   ```
   python prepareGeneListsWholeGenome.py
   ```
3. Create list(s) of exons. Repeat for each list file generated in step 2.

   ```
   python createListExons.py <listFile>
   ```

   `<listFile>` is a file generated by `createListExons.py`. Use only the filename; strip the directory and file extension.
4. Prepare the exon sequences files. Repeat for each list file generated in step 2.

   ```
   python prepareExonSequences.py <listFile>
   ```

   `<listFile>` is a file generated by `createListExons.py`. Use only the filename; strip the directory and file extension.
5. Prepare list of off-target sites

   ```
   python prepareListOfftargetSites.py
   ```
6. We need to create the all\_sequences.txt file (its going to be duplicate of the FASTA file):

   ```
   cp /home/jake/tools/mm10-CRISPR-DB/mm10_input/chr19.fa /home/jake/tools/mm10-CRISPR-DB/mm10_input/all_sequences.txt
   ```

**Code modifications:**

- Line 32 in `prepareExonSequences.py` (from -> to):

  `dir_seq = "./mm10_input/chr_sequences/"`

  `dir_seq = "./mm10_input/"`
- Line 81 in `prepareExonSequences.py` (from -> to):

  `chr = chr_offset[match.group(1)]`

  `chr = 1`
- After line 83 (`end = ast.literal...`), add the following:

  ```
  if len(chrSeq) < end+padding and (start-padding) > 0 and (end+padding) > 0:
    temp = chrSeq[chr][start-padding:end+padding].upper()
    if len(temp) > 0:
        outFile.write(chrSeq[chr][start-padding:end+padding].upper()+"\n")
  ```

We prepared a brief brash script to automate the pipline, `run-tool-sbs.sh`:

```
#!/bin/bash
python prepareListOfftargetSites.py

# Run method on each gene list
FILES=./mm10_input/list_???.txt
for f in $FILES
do
    NAME=${f:13:8}
    python target_identitification_viaC.py nb_threads_C=128 nb_threads_Bowtie=8 genes=$NAME
done
```

**Run:**

```
python /home/jake/sbs/sbs.py -c "./run-tool-sbs.sh" -o /home/jake/sbs/output/mm10-CRISPR-Database-0005/mm10-CRISPR-Database-0005 -l y -s 1 --cmdIsBash
```

---

## CT-Finder

**Version:** 30 July 2015

**Tool specific virtualenv:** No

**Setup:**

- Create a placeholder directory for the JBROWSE\_DATA\_PATH

  `cd /home/jake/crispr/ct-finder && mkdir jbrowse`
- Extend open file limit (https://superuser.com/a/1200818):

  - Modified `/etc/security/limits.conf` with the following lines (this takes care of non-GUI login):

    ```
    jake hard nofile 65535
    jake soft nofile 65535
    ```
  - Logout and log back in
- Files/directories:

  ```
  rm /home/jake/tools/ct-finder/bowtie2db/chr19.fa && ln -s /home/jake/genomes/mm10-ucsc-mod/chr19/500k /home/jake/tools/ct-finder/bowtie2db/chr19.fa

  cd /home/jake/tools/ct-finder/proc && cp -a /home/jake/genomes/mm10-ucsc-mod/chr19/500k/. /home/jake/tools/ct-finder/output/0002 &&
  ```

**Code modifications:**

- In `proc/main.pl` find and replace (from -> to):

  `$ref_genome.'.fa '`

  `$ref_genome.' '`
- Line 790 in `proc/main.pl` (from -> to):

  `system('samtools sort '.$uploaddir.'jbrowse.bam '.$uploaddir.'jbrowse_sorted');`

  `system('samtools sort '.$uploaddir.'jbrowse.bam >> '.$uploaddir.'jbrowse_sorted.bam');`

**Run:**

```
python /home/jake/sbs/sbs.py -c "
perl main.pl Cas9 General /home/jake/tools/ct-finder/jbrowse 0 0 /home/jake/tools/ct-finder/output/0002/ chr19.fa NGG NGG chr19.fa 5 8 1 0 20 1 0 0 2 1 1
" -o /home/jake/sbs/output/ct-finder-0002/ct-finder-0002 -l y -s 1
```

---

## PhytoCRISP-Ex

**Version:** 1.0

**Tool specific virtualenv:** No

**Setup:**

```
cd /home/jake/tools/phytoCRISP-Ex_v1.0/
mkdir install
chmod 755 install.sh
    
./install /home/jake/tools/phytoCRISP-Ex_v1.0/install
    
source /home/jake/tools/phytoCRISP-Ex_v1.0/install/SCRIPTS/crispex_profile

cd /home/jake/tools/phytoCRISP-Ex_v1.0/install/SCRIPTS/
    
chmod 755 phytoCRISPex
    
sudo apt-get install libncbi6-dev
    
cp -a /home/jake/genomes/mm10-ucsc-mod/chr19/500k/. /home/jake/genomes/mm10-ucsc-mod/chr19/500k-phytocrispex

cp /home/jake/genomes/mm10-ucsc-mod/chr19/500k-phytocrispex/chr19.fa /home/jake/genomes/mm10-ucsc-mod/chr19/500k-phytocrispex/chr19.fa.fasta

cd /home/jake/tools/phytoCRISP-Ex_v1.0/install/SCRIPTS/

cd /home/jake/tools/phytoCRISP-Ex_v1.0/
mkdir output-0002
```

To run phytoCRISP-Ex we wrote a brief bash script to automate the pipeline, `0002-sbs.sh`:

```
#!/bin/bash
SCRIPTS/wrapper.sh /home/jake/genomes/mm10-ucsc-mod/chr19/500k-phytocrispex/chr19.fa.fasta /home/jake/genomes/mm10-ucsc-mod/chr19/500k-phytocrispex/chr19.fa.fasta NGG G
```

**Run:**

```
python /home/jake/sbs/sbs.py -c "./0002-sbs.sh" -o /home/jake/sbs/output/PhytoCRISP-Ex-0002/PhytoCRISP-Ex-0002 -l y -s 1 --cmdIsBash
```

**Clean-up:**

```
rm -R /home/jake/genomes/mm10-ucsc-mod/chr19/500k-phytocrispex
```

---

## CRISPOR

**Date obtained:** 31 May 2018

**Tool specific virtualenv:** Yes

**virtualenv packages:**

```
Package                       Version
----------------------------- -------
backports.functools-lru-cache 1.5
biopython                     1.71
cycler                        0.10.0
kiwisolver                    1.0.1
matplotlib                    2.2.2
numpy                         1.14.4
pandas                        0.23.0
pip                           10.0.1
pkg-resources                 0.0.0
psutil                        5.4.6
pyparsing                     2.2.0
python-dateutil               2.7.3
pytz                          2018.4
scikit-learn                  0.16.1
scipy                         1.1.0
setuptools                    39.2.0
six                           1.11.0
subprocess32                  3.5.2
wheel                         0.31.1
xlwt                          1.3.0
```

**Setup:**

- Adjust `bin` directory permissions

  ```
  chmod -R 755 bin/
  ```
- Create a symlink in the genomes directory:

  - Name it: `chr19`
  - Point it to: `/home/jake/genomes/mm10-ucsc-mod/chr19/500k`

**Code modifications:**

- Line 2015 of `crispor.py` (from -> to):

  `score = int(score)`

  `score = int(float(score))`

**Run:**

```
python /home/jake/sbs/sbs.py -c "python /home/jake/tools/CRISPOR/crispor.py chr19 /home/jake/genomes/mm10-ucsc-mod/chr19/500k/chr19.fa guideFileOut" -o /home/jake/sbs/output/CRISPOR-0002/CRISPOR-0002 -l y -s 1
```

---

## CRISPR-DO

**Date obatined:** 23 April 2018

**Tool specific virtualenv:** No

**Setup:**

- Copy the `crisprdo/settings.py.sample` script, rename without `.sample`
- Modify lines 39+ (according to the symlink just created):

  ```
  STATIC_GENOME_2BIT = '/home/jake/tools/crisprdo/genome/{genome}.2bit'
  STATIC_GENOME_CHROM_LEN = '/home/jake/tools/crisprdo/genome/{genome}.sizes'
  STATIC_BWA_INDEX = '/home/jake/tools/crisprdo/genome/{genome}.fa'

  STATIC_DHS = '/home/jake/tools/crisprdo/DHS_{genome}.hammock.gz'
  STATIC_SNP = '/home/jake/tools/crisprdo/{chrom}.bed.gz'
  STATIC_EXON = '/home/jake/tools/crisprdo/{genome}.exon.gz'
  ```
- Modify `line 55`:

  `lasso_dir = '/home/jake/tools/crisprdo/SSC0.1'`
- Modify chromosome mapping (below line 9):

  ```
  genome2species = {'hg19': 'human', 'hg38': 'human',
          'mm9': 'mouse', 'mm10': 'mouse'}
  refdict = {'A': 'T', 'a': 't', 'C': 'G', 'G': 'C', 'g': 'c',
             'c': 'g', 'T': 'A', 't': 'a', 'N': 'N', 'n': 'n'}

  chr19_chroms = ['ucsc-mm10-chr19-full-extract[10000000-10500000]', 
  'ucsc-mm10-chr19-full-extract[10000000-11000000]', 
  'ucsc-mm10-chr19-full-extract[10000000-15000000]', 
  'ucsc-mm10-chr19-full-extract[10000000-30000000]', 
  'ucsc-mm10-chr19-full']

  chrom_lib = {
      'chr19': chr19_chroms
  }
  ```
- Create a soft-link to the input data, add BWA to the OS PATH variable, install CRISPR-DO

  ```
  rm /home/jake/tools/crisprdo/genome && ln -s /home/jake/genomes/mm10-ucsc-mod/chr19/500k /home/jake/tools/crisprdo/genome

  PATH="/home/jake/bwa-master/:$PATH"

  sudo python setup.py install --user
  ```

**Run:**

```
python /home/jake/sbs/sbs.py -c "
crispr-do 
-g chr19 
-c ucsc-mm10-chr19-full-extract[10000000-10500000] 
--start=0 
--end=500000 
--job-id=output-0007
" -o /home/jake/sbs/output/crisprdo-0007/crisprdo-0007 -l y -s 1
```

---

## sgRNAScorer2

**Version:** 2.0

**Tool specific virtualenv:** Yes

**virtualenv packages:**

```
Package       Version
------------- -------
biopython     1.71
numpy         1.14.4
pip           10.0.1
pkg-resources 0.0.0
psutil        5.4.6
scikit-learn  0.19.1
scipy         1.1.0
setuptools    39.2.0
sklearn       0.0
wheel         0.31.1
```

**Code modifications:**

- Insert after line 176 in `identifyPutativegRNASites.V2.py`:

  `outputFile = open(outputFile, 'w+')`
- Update line 215 in `identifyPutativegRNASites.V2.py` (from -> to):

  `parser.add_argument('-i','--inputFASTA',type=argparse.FileType('r'),required=True)`

  `parser.add_argument('-i','--inputFASTA',required=True)`
- Update line 219 in `identifyPutativegRNASites.V2.py` (from -> to):

  `parser.add_argument('-o','--outputFile',type=argparse.FileType('w'),required=True)`

  `parser.add_argument('-o','--outputFile',required=True)`
- Update line 219 in `identifyAndScore.py` (from -> to):

  `outputFile1 = inputFile.name.replace('.fasta','.putative.fasta')`

  `outputFile1 = '%s.out' % inputFile.name`

**Setup:**

```
cd /home/jake/tools/sgRNAScorer2.0/ 

cp /home/jake/genomes/mm10-ucsc-mod/chr19/500k/chr19.fa /home/jake/genomes/mm10-ucsc-mod/chr19/500k/chr19.fa.temp
```

**Run:**

```
python /home/jake/sbs/sbs.py -c "
python identifyAndScore.py 
-i /home/jake/genomes/mm10-ucsc-mod/chr19/500k/chr19.fa.temp 
-o /home/jake/tools/sgRNAScorer2.0/output/0007 
-s 20 
-p 3 
-l NGG
" -o /home/jake/sbs/output/sgRNAScorer2.0-0007/sgRNAScorer2.0-0007 -l y -s 1
```

---

## GuideScan

**Version:** 10 January 2018 (`612b0deb182e71f3c4eaecd70dd167a637113786`)

**Tool specific virtualenv:** Yes

**virtualenv packages:**

```
Package       Version
------------- -------
biopython     1.71
bx-python     0.7.3
guidescan     0.1
numpy         1.14.4
pip           10.0.1
pkg-resources 0.0.0
psutil        5.4.6
pyfaidx       0.4.7.1
pysam         0.8.3
setuptools    39.2.0
six           1.11.0
wheel         0.31.1
xlwt          1.3.0
```

**Setup:**

- Make sure `samtools` is installed

  ```
  cd ~

  git clone https://github.com/samtools/htslib.git

  git clone https://github.com/samtools/samtools.git

  cd ~/samtools

  sudo apt-get install libncurses5-dev libz-dev libbz2-dev liblzma-dev

  make

  nano ~/.bash_profile

  export PATH="/home/jake/samtools/:$PATH"

  source ~/.bash_profile
  ```
- Install guidescan

  ```
  cd ~/tools/guidescan

  python setup.py install
  ```

**Run:**

```
python /home/jake/sbs/sbs.py -c "
guidescan_processer 
-f /home/jake/genomes/mm10-ucsc-mod/chr19/500k/chr19.fa 
-n output-0002 
-l 20
" -o /home/jake/sbs/output/guidescan-0002/guidescan-0002 -l y -s 1
```

---

## FlashFry

**Date obtained:** 2 July 2018

**Tool specific virtualenv:** No

**Setup:**

```
sudo apt-get install default-jdk
```

To run FlashFry, we wrote a brief bash script to automate the pipeline, `0002.sh`:

```
#!/bin/bash
java -Xmx4g -jar FlashFry-assembly-1.8.2.jar --analysis index --tmpLocation ./output-0002 --database ./output-0002/output-0002.index --reference /home/jake/genomes/mm10-ucsc-mod/chr19/500k/chr19.fa --enzyme spcas9ngg

java -Xmx4g -jar FlashFry-assembly-1.8.2.jar --analysis discover --database ./output-0002/output-0002.index --fasta /home/jake/genomes/mm10-ucsc-mod/chr19/500k/chr19.fa --output ./output-0002/0002.discover

java -Xmx4g -jar FlashFry-assembly-1.8.2.jar --analysis score --input ./output-0002/0002.discover --output ./output-0002/0002.scored --scoringMetrics doench2014ontarget,doench2016cfd,dangerous,hsu2013,minot --database ./output-0002/output-0002.index
```

**Run:**

```
python /home/jake/sbs/sbs.py -c "./0002.sh" -o /home/jake/sbs/output/FlashFry-0002/FlashFry-0002 -l y -s 1
```

---

## TUSCAN (offline edition)

**Date obtained:** 24 January 2019

**Run:**

```
python /home/jake/sbs/sbs.py -c "
python TUSCAN.py 
-m Regression 
-g /home/jake/genomes/mm10-ucsc-mod/chr19/500k/chr19.fa 
-c ucsc-mm10-chr19-full-extract[10000000-10500000] 
-s 0 
-f 500000 
-o output-0001.tsv
" -o /home/jake/sbs/output/tuscan_20190124-0001/tuscan_20190124-0001 -l y -s 1
```
